# Oncolytic Virus Engineering and Utilizations: Cancer Immunotherapy Perspective

**DOI:** 10.3390/v15081645

**Published:** 2023-07-28

**Authors:** Palaniyandi Muthukutty, So Young Yoo

**Affiliations:** BIO-IT Foundry Technology Institute, Pusan National University, Busan 46241, Republic of Korea

**Keywords:** oncolytic virus, cancer, immunotherapy, virotherapy, clinical trials

## Abstract

Oncolytic viruses have positively impacted cancer immunotherapy over the past 20 years. Both natural and genetically modified viruses have shown promising results in treating various cancers. Various regulatory authorities worldwide have approved four commercial oncolytic viruses, and more are being developed to overcome this limitation and obtain better anti-tumor responses in clinical trials at various stages. Faster advancements in translating research into the commercialization of cancer immunotherapy and a comprehensive understanding of the modification strategies will widen the current knowledge of future technologies related to the development of oncolytic viruses. In this review, we discuss the strategies of virus engineering and the progress of clinical trials to achieve virotherapeutics.

## 1. Introduction

Cancer is the second leading cause of disease-related mortality and concern worldwide [1]. Even though early detection and increased survival rates have been achieved through precision medicine or immunotherapies, the development of cancer treatments for completely eradicating cancers is still of high priority. An array of technologies has been developed to treat various types of cancers. New insights into molecular and immunology-based research combined with cutting-edge therapeutic approaches, such as monoclonal antibodies, molecular-targeted drugs, and oncolytic viruses, have helped to manage cancer better.

Tumors are conglomerations of cells forming the tumor microenvironment (TME). The TME is a highly complex heterogeneous cell mass consisting of cancer stem cells, endothelial cells, fibroblasts, extracellular matrices, immune cells, connective tissue, blood vessels, and signaling molecules. The TME plays a crucial role in tumor growth, invasion, and metastasis, making it a key target for cancer immunotherapy. However, the TME is populated with immunosuppressive cells, such as regulatory T cells (Tregs), tumor-associated macrophages (TAMs), and myeloid-derived stem cells (MDSCs). Thus, immunosuppressive TME is an important target for therapeutic applications and poses a challenge for cancer immunotherapy. Multiple strategies are employed to overcome the TME [2].

Treatment of cancer using viruses that can be naturally or genetically altered to attack and kill cancer cells can be performed without harming normal cells. During the early 1900s, patients with virus-infected cancer showed tumor remission for a short time, which led to the development of oncolytic viruses (OVs) [3,4,5]. Oncolytic viruses (OVs) are viruses that can disseminate in cancer cells [6]. A better understanding of the molecular biology of viral infections and the advancement of OVs with genetic engineering has led to the development of oncolytic virotherapy [7]. A wide range of OVs, including both DNA and RNA viruses, have been developed for cancer immunotherapy. DNA viruses mainly comprise double-stranded viruses, such as adenovirus (AdV), vaccinia virus (VACV), and herpesvirus (HSV), whereas RNA viruses are single-stranded RNA viruses, including the positive sense strand [Coxsackievirus (CSV), poliovirus (PV), Seneca Valley virus (SVV)] and negative sense strand [vesicular stomatitis virus (VSV), measles virus (MeV), and Newcastle disease virus (NCDV)].

OVs can be classified into attenuated natural viruses and genetically engineered viruses, some of which are listed in Table 1. These viruses significantly impact OV therapy (OVT) in cancer treatment. OVs have many advantages over conventional immunotherapies, including precise targeting, effective killing rates, and minimal adverse reaction [8,9,10]. For the development of oncolytic virotherapy, both DNA and RNA viruses were manipulated, and their selection process does not follow any standard methods and the range of selection varies from the virus having natural tropism and preferential replication in tumor cells by genetic modification [11].

When considering genetic modification of oncolytic viruses, certain genes are considered as non-essential, and deletion of certain genes can improve the pathogenicity of viral infection and promote virus replication. Also, viruses possessing large genomes can accommodate eukaryotic genes when non-essential genes are deleted. Strategies to manipulate the virus genes include killing the tumor cell by cytotoxicity, activating the immune system, tumor neo-angiogenesis inhibition, and arming with immune stimulatory genes [12].

With the latest understanding and use of viral molecular and cellular interactions, one can develop these disease-causing pathogens into a therapeutic arsenal for invoking the immune system against cancers. OVs are modified transgenically to increase their efficiency by mutating the genes that are essential for their replication in normal cells, manipulating their entry process, increasing their tumor-specific tissue tropisms using specific tumor promoters, and increasing their potential to infect the tumor by inserting genes that can specifically lyse or activate the immune system [4].

OVs can specifically target cancer cells and can be engineered to express transgenes that effectively use four different mechanisms: oncolysis, vascular collapse, anti-tumor immunity, and expression of therapeutic transgenes to counteract cancer cells [13]. Although OV-based therapies have advantages over other cancer therapies, there are certain hindrances to translating them to a commercial scale [14]. The major problem is the delivery of the OV because systemic administration of OVs is a challenge, and one needs to overcome poor target distribution, existing specific antiviral immunity, and innate immune responses [15]. In addition, OVs alone cannot achieve clinical efficacy as its combination with other therapies enhances therapeutic effectiveness. Overcoming these shortcomings could put OVs into the limelight for cancer immunotherapy applications [16].

In this review, we briefly examine the anti-tumor mechanism of OVs, focusing on the many modification tactics used to boost their efficacy through synergistic anti-tumor therapy modalities and transgenic technology. Based on the current regulatory approval of OV-based therapies and preclinical and clinical evidence, we also review the potential applications of OVs in cancer immunotherapy and emphasized the unique difficulties in developing OV therapies. 

**Table 1:** Oncolytic viruses used for cancer immunotherapies and their properties.

	Adenovirus	Vaccinia Virus	Herpesvirus	Reovirus	Poliovirus	Coxsackievirus	Seneca Valley Virus	Measles Virus	Vesicular Stomatitis Virus	Newcastle Disease Virus
Genome	dsDNA	dsDNA	dsDNA	dsRNA	ssRNA(+)	ssRNA(+)	ssRNA(+)	ssRNA(−)	ssRNA(−)	ssRNA(−)
Size	36 kb	190 kb	150 kb	123 kb	7.5 kb	28 kb	7 kb	16 kb	11 kb	15 kb
Capsid	Icosahedral	Complex	Icosahedral	Icosahedral	Icosahedral	Icosahedral	Icosahedral	Icosahedral	Helical	Helical
Virion	Naked	Enveloped	Enveloped	Naked	Naked	Naked	Naked	Enveloped	Enveloped	Enveloped
Access Mechanism	CD46, CAR	Receptor-mediated endocytosis	Nectin-1, Nectin-2, HVEM	Junctional adhesion molecule A (JAM-A)	CD155	CAR/ICAM1/DAF	Endocytosis	SLAM, CD46	LDLR	Sialic acid
Site of Replication	Cytoplasm and Nucleus	Cytoplasm	Cytoplasm and Nucleus	Cytoplasm	Cytoplasm	Cytoplasm	Cytoplasm	Cytoplasm	Cytoplasm	Cytoplasm
Surpassing Blood–Brain Barrier	No	No	No	Yes	Yes	No	Yes	No	No	Yes
Merits	Easy manipulation of genome, production of high viral titers	Well-known virus; large gene modifications, fast replication, and easily manufactured in high viral titers	Multiple transgenes can be inserted into the big genome	Low toxicity, systemic injection applicable	Clinically well-studied virus	systemic injection applicable	Non- infectious and safe for humans	Clinically well-studied virus	Fast replication, non-infectious and safe for human	Non- infectious and safe for human
Demerits	High tissue tropism	Can reverse to infectious virion	Can cause infection; innate virus neutralization	Inadequate gene manipulation	Can reverse to infectious virion	Can reverse to infectious virion, innate virus neutralization	No significant clinical trial output	Can reverse to infectious virion	Inadequate gene manipulation, no clinical trial output	Inadequate gene manipulation
References	[17]	[18]	[19]	[20]	[21]	[22]	[23]	[24]	[25]	[26]

Abbreviations: dsDNA, double-stranded DNA; dsRNA, double-stranded RNA; ssRNA, single-stranded RNA; CAR, coxsackie adenovirus receptor; HVEM, herpes virus entry mediator; JAM-A, junctional adhesion molecule A; ICAM-1, intercellular adhesion molecule 1; DAF, decay-accelerating factor; SLAM, signaling lymphocytic activation molecule; LDLR, low-density lipoprotein receptor; nAbs, neutralizing antibodies.

## 2. Mechanism of Action

The anti-tumor mechanism of OVs involves oncolysis of the infected tumor cells, and the infection mechanism depends on the type of virus infecting the tumor cells. The susceptibility of the tumor to cell lysis can influence the efficacy of the virus. In addition, the amplification and spread of OVs depend on the antiviral immune response of the host immune system, which determines the success of oncolytic virotherapy. By stimulating the recruitment of immune cells and activating systemic anticancer adaptive immunity to limit tumor growth, OVs stimulate innate immunity and transform “cold” tumors into “hot” tumors [27,28,29]. After adhering to and invading tumor cells, OVs can use several lytic pathways, some of which may or may not be related to viral replication within the target cells. The virus overtakes the tumor cell’s protein translational machinery to inhibit protein synthesis, destroying tumor cells. The production of viral nucleic acids and proteins results in the formation of progeny virus particles released through cell lysis and the destruction of tumor cells [30]. The immune system detects tumor cells after the lysed cells release cytokines and chemokines, which help induce subsequent pathways to detect tumor cells. When the tumor undergoes apoptosis, tumor-derived antigens are released, which attract cytotoxic T lymphocytes, dendritic cells, natural killer cells, and macrophages to induce a tumor-specific immune response, resulting in the recognition and coordinated attack of tumor cells [31,32].

Cancer cells infected by OVs undergo cell death through various cell-death pathways, such as apoptosis, necrosis, autophagy, ferroptosis, and pyroptosis-based disintegration, leading to the exposure of antigens to the immune system present inside the TME, which is known as immunogenic cell death (ICD). With respect to OVs pathophysiology, ICD plays a key role in promoting anti-tumor immunity [33,34]. The ICD of OVs releases tumor-associated antigens (TAAs), damage-associated molecular patterns (DAMPs), and pathogen-associated molecular patterns (PAMPs), and the upregulation of multiple inflammatory cytokines activates both innate and adaptive immune responses [35,36,37]. Furthermore, there is an immune-response-associated bystander effect in which the release of cytokines from lytic tumor cells can attract an immune response to neighboring tumors without the lysis of that particular tumor. OVs can also disrupt the blood vessels connected to tumors, leading to a lack of oxygen and nutrients, causing the tumor to weaken [38]. One of the main obstacles for the immune system to target tumors is the immunosuppressive TME. OVs provide the platform wherein the immunologically “cold tumors” are converted by oncolysis to “hot tumors” [39,40,41]. OVs overcome the immunosuppressive environment by targeting immune-modulating genes of immune checkpoint inhibitors (ICI), tumor antigens, and chimeric antigen receptor T cells [42]. However, solid tumors are heterogeneous, complex structures that are imperative for the penetration of OVs into tumors and hamper their function. To overcome these obstacles, it is crucial to strategize one or more modes by which OVs can penetrate the solid tumors or how they can be combined with other immunostimulatory molecules that can penetrate the TME or promote anti-tumor immune responses. This demonstrates that OVs, in combination with existing therapeutics, can mutually suppress tumor growth and effectively eliminate cancer cells.

Several strategies are used in the genetic engineering of OVs to enhance the potency of the virus to effectively replicate and kill the tumor cells. One such tactic involves modifying the extracellular matrix (ECM) to enhance virus dissemination into primary tumor and nearby secondary tumor sites. Several studies have modified adenovirus to encode relaxin to degrade the matrix metalloproteinases (MMPs), which in turn degrade the ECM [43,44]. To increase tissue permeability, oncolytic adenovirus was modified to encode hyaluronidase to target hyaluronan in the ECM [45]. Other strategies by which the oncolytic virus can alter the mechanism, such as adding endonuclease DNase I into the oncolytic adenovirus, could eliminate the free DNA and enhance virus spread [46]. Oncolytic HSV expressing vasculostatin-120 is able to disseminate virus and control vascularization of cancer tissues [47], and several other studies have modified the OV mechanism by altering tumor cell signaling [48,49,50,51,52]. Genetic engineering techniques have been used to manipulate oncolytic viruses that shows cancer-selective replication and proliferation and improved anti-tumor activity.

## 3. Strategies with OVs

Each cancer cell acts differently, and each OV acts differently regarding penetration and replication in different cancer cells. Many modification strategies have been implemented to improve OV rights by increasing the binding capacity and target accuracy, viral replication, and the capacity to disseminate tumor cells. One of the challenges oncolytic virotherapy faces is the suppressive tumor microenvironment that inhibits T-cell activity and supports tumor progression, limiting its therapeutic benefits to a restricted fraction of patients treated with immunotherapy. Additionally, new immunotherapeutic treatments have led to new adverse immunological events, including cytokine storms and autoimmune events. Another challenge is the need to better understand the individual immune environments to provide maximal patient benefits.

**Figure 1:**
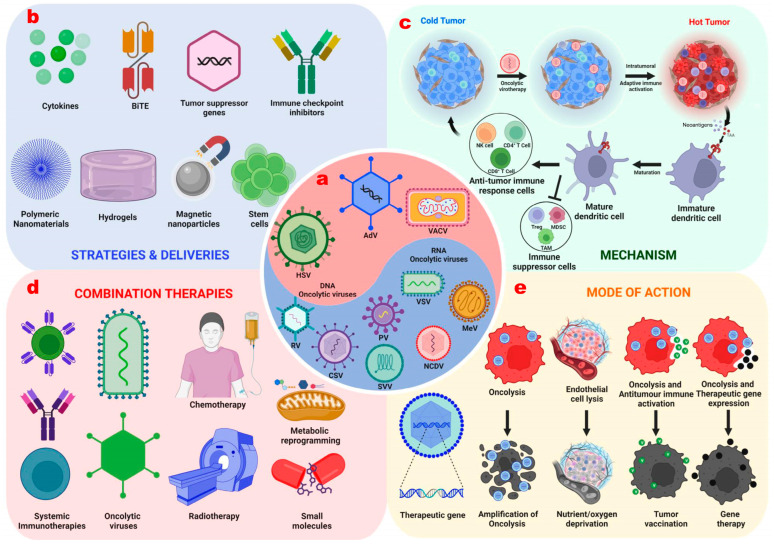
Strategies involved in enhancing the therapeutic potential of Oncolytic Virotherapies. Strategic approaches to enhance the novel OVs by genetic engineering by combining them with other therapeutics and by enhancing efficient delivery modalities. These approaches aim to help the OVs achieve a higher anti-tumor mechanism, resulting in various anti-tumor activities and overall tumor reduction. (**a**) DNA and RNA viruses are used for genetic manipulation to form oncolytic viruses; (**b**) Modalities by which OV are manipulated and delivered to the tumor site; (**c**) Oncolytic virus converts immunologically “Cold” tumor into “Hot” tumor by oncolysis and immunological anti-tumor activities; (**d**) To enhance the effectiveness of OV by combining with traditional therapies; (**e**) These genetic modification and combinations can lead to the destruction of tumor cells by various modes. OV, oncolytic viruses; NK, natural killer cells; MDSC, myeloid-derived stem cells; TAM, tumor-associated macrophages; AdV, adenovirus; HSV, herpes simplex virus; VACV, vaccinia virus; RV, reovirus; CSV, coxsackie virus; PV, poliovirus; SVV, Seneca Valley Virus; MeV, measles virus; NCDV, Newcastle disease virus; VSV, vesicular stomatitis virus; BiTE, Bi-specific T-cell engager.

There are several limitations to OVT. One of the main limitations is the lack of virus specificity, which can lead to off-target effects and damage healthy cells. Another limitation is the development of antiviral immunity, which limits the effectiveness of treatment. Additionally, the tumor microenvironment can be immunosuppressive, limiting the ability of the immune system to mount an effective viral response. Finally, the efficacy of oncolytic virotherapy is limited by the ability of the virus to penetrate the tumor and infect cancer cells. To overcome these shortcomings, efforts have been made to optimize the effectiveness of viruses against the immune system (Figure 1). 

### 3.1. Genetic Engineering of OVs

Developing safe cancer-selective and highly effective OVs against various tumors relies heavily on the genetic engineering of OVs [53,54]. Understanding the biology and genetics of the virus, interactions between the virus and the host, how infected cells die, and how cells defend themselves from lytic infection are crucial for any modification of OVs [27]. Genetic engineering using different transgenes has enabled OV application in cancer immunotherapy to broadly activate the anti-tumor immune response, enhancing OVs tumor cell tropism and reducing toxicity to normal cells [55]. They can be categorized into several groups according to the function and type of transgene used for OV engineering and alterations [56].

The insertion or substitution of proteins in viruses can be performed for tumor targeting. One such study introduced a glycoprotein variant of the lymphocytic choriomeningitis virus (LCMV) by substituting the VSV glycoprotein G for selective replication in cancer cells. Mutation or deletion of the thymidine kinase (TK) gene in VACV and HSV-1 or E1B55K in oncolytic AdV ONYX-015 allows OV to selectively replicate in cancer cells [4,57,58]. The study performed deletion or modification of viral genes that improve replication in tumors without harming healthy tissues. In this case, they selectively deleted the ICP34.5 and ICP47 genes of HSV-1, which can selectively replicate in cancer cells [59,60,61,62]. OVs express immunostimulatory cytokines and chemokines. Cytokines such as GM-CSF, IL-12,15,18, 23, 24, 36γ, TNF, and IFN-a/b can enhance the anti-tumor response, and tumor lysis has been demonstrated in several studies and clinical trials [63,64]. The expression of these cytokines in the tumor microenvironment can effectively reduce toxicity when systemically infused for cancer immunotherapy.

A commercially available OV product, T-VEC, is genetically engineered to express GM-CSF and enhance the recruitment of antigen-presenting cells (APCs) to the immunosuppressive tumor microenvironment, which leads to the recruitment of other cytokines and enhances anti-tumor activity [65,66]. IL-12 expressed in APCs can stimulate T helper cells; moreover, it has been used in many OV manipulations and has shown potent anti-tumor activity in many clinical studies [67,68,69,70,71,72]. Chemokines are chemotactic cytokines that mediate immune cell migration. Chemokines, such as CCL2, 5, 20, 21, 22, CXCL10, 11, and CLL19, have been used to engineer OVs aiming to attract immune cells to tumor sites. Different immune cell subsets migrate to the TME in response to chemokine stimulation, where they spatiotemporally influence anti-tumor immune responses [73].

In one study, VACV expressing CXCL11 delivered intratumorally induced the aggregation of T cells in tumor tissues and increased the survival of tumor-bearing mice [74,75,76]. In a mouse colon cancer model, the combination of OVT with a chemokine-enhancing cocktail that increased CCL5 and CXCL10 production while reducing CCL22 was demonstrated to boost the trafficking of T helper cells and CTLs to the TME and improve survival [77]. In another study on colon cancer and neuroblastoma, the infusion of viruses carrying CCL5 or CCL2 enhanced the number of Th1 infiltrating cells in the TME [78,79,80]. NG-641, an oncolytic AdV expressing CXCL9, CXCL10, and IFNA, is undergoing clinical trials. In many other preclinical studies, OVs expressing chemokines have also demonstrated improved effectiveness [78,81]. These studies demonstrated that using OVs armed with chemokines can effectively and efficiently increase T-cell infiltration into the tumor.

### 3.2. Modification to Improve Specificity to Tumors

Other strategies include introducing tumor-specific antigens that help OVs activate systemic immunity using a genetic engineering approach. The OVs can be modified by adding TAA, further enhancing anti-tumor immunity [82,83]. Bilusic et al. conducted a phase I study using multitargeted AdV 5 vectors constructed with three TAAs, namely prostate-specific antigen (PSA), brachyury, and MUC-1, and developed a cancer vaccine against castration-resistant prostate cancer; they reported that the patients showed a multifunctional T-cell response to TAAs [84]. Bi- or Tri-specific T-cell engager (BiTE or TriTE) antibodies are another strategy for modifying OVs. Using this technique, two different antibodies of single-chain fragment variables (ScFVs) are linked so that each fragment can attach itself to both T cells and the surface of malignant cells [85,86,87]. Yu et al. showed vaccinia virus armed with BiTE made up of two single-chain variable fragments for CD3 and tumor cell surface antigen EphA2 had potentially killed tumor cells; they also showed an abscopal effect by T-cell-mediated activation and tumor lysis [88]. Several other OVs armed with BiTE, such as FAP and EGFR, have proved to enhance T-cell activation and accumulation in the tumor site, resulting in higher anti-tumor efficacy [89,90,91]. In a study using BiTE and TriTE, an armed oncolytic AdV showed a reduction in tumor-associated macrophages in samples from patients with cancer [92]. OV-encoding BiTE increases T-cell infiltration into the TME and enhances anti-tumor activity with less toxicity [93]. In a recent study, HSV-1 expressing programmed cell death receptor (PD-L1) BiTE exhibited anti-tumor activity by enhancing T-cell activation and cytokines against immunologically cold tumors [94].

### 3.3. Combination Strategies for OVs

To enhance the potential of OVTs, several supplementary approaches have been combined to compensate for their disadvantages [95,96,97]. Costimulatory molecules, such as CD28 and B7.1, intercellular adhesion molecule 1, and ICIs, have significant roles in cancer immunotherapy. The immune system has checkpoint-inhibiting proteins that prevent interaction with the counter protein from overcoming the immunosuppressive activity in the TME, whereas cancer cells take over this mechanism to prevent anti-tumor immunity [98,99]. The development of monoclonal antibodies that can target these checkpoint inhibitors has been demonstrated in studies of solid tumors [100,101].

In the body, tumor cells often exhibit properties such as evasion of immune surveillance and loss of immunological response. Consequently, cancer spreads faster and has a worse prognosis in patients with higher levels of malignancy. These biological characteristics of tumor cells are related to the host immune system, such as PD-L1 and programmed cell death protein 1 (PD-1), in proportion to the upregulation of cytotoxic T-lymphocyte-associated protein 4 (CTLA-4) [102,103]. However, ICI-based studies exhibit limited success due to their restricted effect on cold tumors because of the low tumor-infiltrating lymphocyte (TILs), leading to a low response rate (10–20%) in patients with cancer and due to some adverse events; thus, ICIs may have better effects when used in combination therapies. To compensate for this, we must combine checkpoint inhibitors with OVs, which have shown promising results based on research and preclinical studies [104,105,106,107,108,109]. ICIs, such as anti-PD1, anti-PDL1, or anti-CTLA4, are engineered to express these antibodies in the tumor itself so that they can be less toxic than their systemic delivery [103,108,110,111,112,113,114]. This provides a potential approach to treating cancer by combining ICIs with virotherapy [106].

Currently, clinical trials combining OVs with ICIs include Pexavac VACV with anti-PDL1 and anti-CTLA4, LOAd703 AdV with anti-PD-L1, HSV-1 OH2 with anti-PD1, and RV Reolysin with anti-PD1; furthermore, some clinical trials are in various phases of their treatment [115]. The use of genetically engineered OVs expressing checkpoint-inhibiting antibodies has proven to have a synergistic effect on the TME with the least side effects [116,117,118]. OVs are genetically engineered to express cytokines possess value in addition to the combination of ICI and have enhanced the efficacy of OV treatments [119,120,121].

### 3.4. Delivery Innovations for OVs

One of the key factors determining the effectiveness of any drug is its effective delivery to the tumor site without harming normal cells. Common delivery methods include intratumoral injection, intravenous injection, and site-specific delivery of tumors to visceral lesions under ultrasound guidance, which are comparatively complicated and pose risks such as infection-related complications [122]. To improve efficacy, one must design a delivery system that can home the tumor without degradation and release the drug into the tumor site. One has to adopt a strategy by which the OVs are able to surpass the immune system and effectively deliver the OV when injected by intravenous infusion; recently, inhibition of phosphatidylinositol 3-kinase δ (PI3Kδ) showed it was able to protect the OV from macrophages, which in turn enhanced the anti-tumor efficacy of intravenous delivered OVs [123]. Several studies have been conducted to deliver the OV specifically to the tumor site. The bioengineered cell membrane nanovesicle (BCMN) is a novel systemic delivering OV to the cancer site that was designed by Peng et al., in which the BCMN can wrap around the oncolytic adenovirus showing prolonged circulation time and increased survival rate [124]. In another study, coating oncolytic adenovirus with serum albumin can prevent the virus from neutralizing antibodies [125]. Badrinath et al. enhanced oncolytic vaccinia virus anti-tumor efficacy by increasing its apoptosis using poly lactic-co-lactic glycolic acid nanofiber as a delivery method against colon carcinoma [126]. Various chemical and biological payloads have been extensively studied to efficiently protect, home, and deliver OVs [16,127,128,129].

Chemical delivery methods include the use of chemical-based polymers such as PEG, hydrogels (natural and synthetic), nano-biomaterial-based metal nanoparticles, liposomes, polymeric micelles, and biological carriers such as stem cells, natural killer (NK) cells, and chimeric antigen receptor (CAR) T cells; however, few delivery approaches were tested in clinical trials [130,131,132,133,134,135]. Physical methods include magnetic nanoparticle-encapsulated OV delivery, which effectively enhances the infection rate, leading to tumor suppression [136,137]. An ultrasound-guided acoustic cavitation-based delivery mechanism has been used for VACV delivery, targeted drug release, and retardation of tumor growth [137,138].

In one study, encapsulation of oncolytic adenovirus with a nanocarrier polygalactosyl-b-agmatyl diblock copolymer against hepatocellular carcinoma showed high affinity towards liver surfaces [139]. In another study, engineered oncolytic adenovirus carrying calcium and manganese carbonate biomineral shells (MnCaCS) helped oncolytic adenovirus from detection from immune system, leading to extended circulation in the blood and an improvement in the replication ability of the virus [140].

Biological vehicles are another delivery strategy for protecting OV [141]. Stem cells have been used as delivery modules for OV [142,143]. One advantage of using stem cells is that the systemic administration of OV causes high levels of homing, even at a low infectious dose [144]. Na et al. developed a novel systemic delivery of oncolytic adenovirus (OA) expressing relaxin into MSC as delivery vehicle against pancreatic tumor and showed a higher infiltration rate and tumor tropism [145]. In another study, when menstrual-blood-derived MSC was combined with ICOVIR15-cBiTE, the anti-tumor effect was higher compared to the control groups [146]. In a pancreatic ductal adenocarcinoma (PDAC) study, genetically modified myxovirus (encoding tumor necrosis factor ligand superfamily member 14) loaded into adipose-derived MSC showed effective delivery and tumor regression [147]. With the development of Chimeric antigen receptor-redirected T cells (CAR-T cells), adoptive T-cell therapies (ACTs) have been extensively studied for their anti-tumor activities. Combining CAR-T-cell therapy with OVs has been shown to increase the efficacy against solid tumors [148]. Recently, an animal model treated with a combination of cell carrier CAR-T and OA delivered systemically with MSC showed anti-tumor immunity in cancer patients [143].

### 3.5. Combination of OVTs with Radiotherapy and Chemotherapy

Multiple anticancer mechanisms can act directly or indirectly on tumors, making OVs an appropriate strategy for cancer immunotherapy. In addition, they can be genetically engineered and have proven safety standards because of the versatility of OVs used in various natural or genetic engineering approaches, and they have been tested in clinical trials [117]. This synergistic effect has been tested in combination with chemotherapy, radiotherapy, targeted therapy, and immunotherapy.

When OVs are combined with radiotherapy, both techniques produce synergistic anti-tumor effects and enhance immune responses against aggressive tumors [149,150,151,152,153]. A study was conducted using the OV Delta-24-RGD combined with radiography for pediatric high-grade gliomas (pHGG) and diffuse intrinsic pontine gliomas (DIPGs), models 166. In another study, an OV vesicular stomatitis virus expressing IFNβ (VSV-IFNβ) combined with radiotherapy exhibited improved anti-tumor activity and tumor reduction in a syngeneic model [154]. Chimeric AdV type 11p (Enadenotucirev), radiation, and chemotherapy (capecitabine, a non-cytotoxic precursor to 5-fluorouracil) are currently being tested in a phase I clinical trial for locally advanced rectal cancer (NCT03916510). Detailed combinatorial research has been explored in detail in recent reviews [155].

### 3.6. Combination with Cell Therapy

Cellular immunotherapy, commonly referred to as adoptive cell therapy, uses altered immune cells to eradicate cancer cells. Various cellular immunotherapies have been developed, including tumor-infiltrating lymphocyte (TIL), engineered T-cell receptor (TCR), CAR-T cells, and NK cell therapies. Of these, CAR T-cell therapy has shown the highest efficacy against blood cancers. However, cell therapies have limitations with respect to solid tumors, owing to their poor infiltration capacity. A combination of cellular therapies with OVs, especially engineered OVs, such as AdV expressing IL-7 combined with B7H3-targeted CAR-T cells, showed enhanced efficacy compared to the use of individual therapies [156]. Another study using a combination of cytokines, ICIs, and BiTE molecules accompanied by HER2-Specific CAR-T cells significantly improved anti-tumor effectiveness, tumor reduction, and overall survival [157].

## 4. Approved OVs against Cancers

Several viruses with good potential for cancer treatment are currently being developed for cancer virotherapies. The development of initial proof-of-concept studies for clinical trials has led to the development of several FDA-approved commercial OVs for cancer immunotherapy (Table 2). Even though clinical trial studies support the therapeutic potential of OVs, one has to consider several aspects during the trial process. Choosing the viral species based on the tropism, performing modifications using genetic engineering, choosing the route and timing of administration, and looking into patient demographics and the potential immune response are a few parameters that must be considered.

Since OVs have multiple mechanisms of action, one has to give importance to safety by clinical validation through statistically proven clinical trials [158,159]. ONYX-015, also known as d11520, was the first proof-of-concept clinical trial for OVs conducted in 1997. ONYX-015 clinically proved its safety and effectiveness in treating patients with head and neck cancer, and its anti-tumor efficacy is further enhanced when combined with chemotherapies [160,161,162].

The first OV, Rigvir, was approved in 2004 by Latvia for the treatment of melanoma. Rigvir is approved for treating melanoma and is composed of genetically un-modified *Picarnoviridae* family *Enterovirus* genus (ECHO type 7) viruses [163]. In a retrospective study, Rigvir showed a significantly lower mortality rate of 4.39–6.57-fold in patients with melanoma stage IB, IIA, IIB, and IIC with no adverse effects [164]. The route of administration was through intramuscular injection, and the study did not show statistically significant results compared with the control group; however, a higher survival rate was observed in patients who underwent Rigvir treatment. Despite receiving regulatory approval, few studies have described its biological properties and effectiveness in treating malignant tumors. Only limited publication data are available for the efficacy of Rigvir; one is a case report of three patients, and the other is a retrospective study on early-stage melanoma. A case study reported that the patient was treated with Rigvir after surgery [165].

In 2005, a second OV was approved in China; the oncolytic AdV H101 was approved for nasopharyngeal carcinoma of head and neck cancer. In this OV, deletion of E1B-55kD and partial deletion of E3 from an AdV combined with chemotherapy showed enhanced anti-tumor activity compared to the control group [166].

Subsequently, Talimogene laherparepvec (T-VEC) was approved by the US FDA in 2015 for the treatment of melanoma. T-VEC is a modified HSV-1 virus with deletions of IC 34.5 and ICP47 that can enhance the tumor-suppression activity and safety of normal cells, and the transgene GM-CSF promotes APCs and induces systemic anti-tumor immunity. T-VEC is an intratumoral mode of administration injection, which works on the dual mechanism of action of oncolysis of tumors with the secondary function of the systemic immune response. Infection and replication of the herpes virus lead to the lysis of tumors, and the release of soluble tumor-associated antigens and tumor cell debris activates the local and systemic immune systems to act on the immunosuppressive TME. In contrast, the GM-CSF can trigger APCs, such as dendritic cells, which can present tumor antigens to specific T cells, triggering a systemic immune response. Several preclinical and clinical studies have demonstrated the efficacy, effectiveness, and safety of HSV-1 as an OV in cancer immunotherapy [65,167].

Recently, Japan approved a third-generation recombinant HSV-1 OV named Teserpaturev (G47∆) (Delytact) for glioma 133. G47∆ is a triple-mutated oncolytic HSV-1 in which the deletion of ICP 34.5 and ICP 47 and lacZ inactivation could potentially enhance tumor specificity, virus replication, and sustained immune response 134. It is an intratumoral injection, and the phase II study showed that the one-year survival rate was 84.2% for the higher dose, and the median overall survival rate was 28.8 months from the G47∆ initiation. This OV has also been clinically tested for other cancer treatments, such as esophageal cancer, prostate adenocarcinoma, tongue cancer, metastatic breast cancer, and gastric cancer, with promising results [168].

Even with all these approved OVs hitting the market, their efficacy is still limited to locoregional lesions than in distant tumors. The recently approved G47∆ for glioblastoma can more effectively combat treated lesions than the distant tumors. Even the approved T-VEC efficacy as a monotherapy is not very comparable with traditional chemotherapy or ICI in case of melanoma. These limitations have put OVs in combination with ICIs or traditional therapies or with surgeries and have shown extremely significant therapeutic advantages [169].

Oncolytic virotherapy efficacy can be enhanced by identifying the histologic tumor type, cancer cell parameters, such as the receptors and genetics of the cell, and patient immune status. Increased efficacy can be achieved with potent virus species or strain, which can be manipulated in arming the transgene and has to be well adopted for combinatorial regimens. Overcoming the induction of tumor-specific immunity by maintaining immune-modifying genes that evade the immune responses are crucial in designing OVs. Also, non-genetic approaches such as coating the viruses with polymers are able to evade the physical and blood barriers during circulation. At this time, there is more anticipation to improve the potency of the OVs in clinical trials that give further insights into the future in oncolytic virotherapy [127,155,170,171].

## 5. Recent Clinical Trials for OVTs

Although the idea of OVs has been around for nearly a century, in the last 40 years, OVs have been intensively researched, and preclinical studies have supported the concept and efficacy of OVT [3,172]. With the efficacy and safety well established after the first approval of oncolytic virotherapy in 2004 (Rigvir), various viruses have been extensively researched and genetically engineered to enhance their effectiveness against various surface and solid tumors. The major viruses studied are AdV, VACV, HSV-1, and HSV-2, along with other viruses showing potential, including CSV, NCDV, and reovirus (RV). In the past five years, from phase I clinical trials, clinical data have been published with knowledge limiting safety. The general perception is that the OVs are generally safe; in all of those safety studies, adverse effects were limited to grade ≤3 and were observed for OVs with higher objective response rates (ORRs) [173,174]. As of 2 June 2023, 106 studies had undertaken OV in various trial phases, of which 33 studies were completed, according to ClinicalTrials.gov. In these trials, 31 OV products were used to treat various cancers, with most studies being in the early stages and only a few in the phase III stage. Nearly 30 different tumors are treated by OVs registered for clinical trials, including melanoma, liver cancer, head and neck cancer, glioma, bladder cancer, pancreatic cancer, nasopharyngeal cancer, and lung cancer [175]. Some of the ongoing clinical trials are listed in Table 3.

### 5.1. Oncolytic Adenovirus (oAds)

Oncolytic adenovirus (oAds) is one of the earliest candidates as OVs investigated in clinical trials for oncolytic virotherapy against cancer treatments. In 2005, Oncorine was first approved in China, and it proved to be effective with oncolytic adenovirus in a clinical trial, showing strong anti-tumor activity, anti-angiogenic effects, increased transgene expression [166]. But it has failed to show that administering it alone would be effective enough against refractory solid tumors. Strategies to maximize the generation of a systemic anticancer immune response that is mediated by oAds may be a more possible way to optimize the anti-tumor effect of oAds in the non-injected lesions, even though systemic administration of oAds remains a significant barrier [201]. Currently, there are two clinical trials ongoing using oAds expression GM-CSF (CG0070 and ONCOS-102) with promising results against solid tumors [202,203]. A phase 1/2a clinical trial LOAd703, expressing CD40L and 41BBL to stimulate the respective pathways will in turn induce the anti-tumor immune response [204]. Systemic delivery of oAds poses a major challenge, but strategies that are devised to increase the systemic anti-tumor immune response by oAds could effectively increase the anti-tumor effect of oAd in the non-injected lesions. There is an increasing number of trials examining oAds armed with pro-inflammatory immune transgenes in order to maximize the viruses’ ability to trigger a strong systemic anti-tumor immune response, which is a crucial factor in controlling the growth of non-injectable and metastatic lesions in patients with advanced stages of cancer [205,206].

### 5.2. Oncolytic Herpes Simplex Virus (oHSV)

Like numerous other OV types, oHSV has the ability to directly destroy tumor cells while triggering an anticancer immune response. As one of the few viruses with well-established antiviral medications, HSV is treatable by clinicians with the existing knowledge and training [207]. oHSV almost infects nearly all cancer types, which is advantageous in clinical settings where variability of tumors and corresponding phenotypic changes require adaptability and broad target coverage to elicit the most effective achievable therapeutic response [208]. As discussed above T-VEC was the first OV approved by the FDA and EMA for its anti-tumor effect against melanoma patients. A third-generation oHSV based on G207 called G47∆ has been the subject of substantial clinical research in Japan; it has recently received conditional approval for the treatment of patients with malignant glioma or any primary brain cancer. These success makes oHSV an ideal candidate for many other clinical trials incorporating transgenic cytokines for various cancer ailments. One major challenge with oHSV is their presence outside the tumor, which could possibly be overcome by increasing their specificity to tumor-specific tropism, increased tumor infiltration, and sustainable anti-tumor activities in the TME could improve the potentiality of oHSV against various cancers [209].

### 5.3. Oncolytic Vaccinia Virus (oVV)

oVV has shown its effectiveness by infecting, replicating, and killing various cancer cells. Its large genome size can accommodate large transgenes with a high safety profile [18]. The extensively clinical trialed oVV is Pexa-Vec (JX-594) expressing GM-CSF transgene. Pexa-Vec had an excellent safety profile and was well-tolerated by patients with refractory solid tumors (NCT01169584) [210]. Another phase I trial using IV administration of JX-929 to treat solid tumors (NCT00574977) showed an excellent safety profile with no dose-related toxicity or severe side events [211]. Because systemic administration cannot sufficiently deliver oVV to tumor tissues to induce a therapeutic effect, these clinical trial results of oVV therapy clearly revealed that intratumoral injection of oVV should be the preferred mode of administration in future clinical trials [212].

Clinical trials have shown that OVs are typically administered as local injections in combination with other therapies, including chemotherapy or ICIs. Therefore, it is possible to hypothesize that combination therapy is a vital modality for OVs to play a significant role in the anti-tumor field. Furthermore, it can be postulated that combination therapy is crucial for OVs to play a significant role in anti-tumor therapies. Currently, intratumoral injection or infusion into the cavity is the primary method for administering OVs, resulting in a local concentration of OVs.

Clinical studies have revealed that oncolytic virotherapy has a distant tumor-suppressive effect; however, the effects in the lesions receiving direct injection are superior to those in distal lesions [63,168]. Another development that must be made with OVs for cancer immunotherapy is to deliver the OVs through intravenous routes, as direct delivery to the lesion is difficult for certain types of cancer. The intravenous administration of Pelareorep, a serotype 3 RV, has demonstrated acceptable tolerability and encouraging efficacy in treating advanced pancreatic cancer and melanoma.

In colorectal cancer, breast cancer, and non-small-cell lung cancer (NSCLC), no progression-free survival has been reported in a randomized phase II trial [213,214,215,216,217]. Overall, clinical trials showed that intratumoral injection showed significant improvements compared to intravenous injection [174]. Future clinical trials are needed to improve the effectiveness of intravenous delivery of OVs. Thus, research studies attempting to overcome these challenges are required to boost therapeutic concentrations of OVs with systemic delivery, low toxicity, and improved efficacy.

## 6. Challenges and Future Prospects

There is overwhelming evidence of oncolytic virotherapy as a substantial advancement in cancer treatment with durable and effective clinical outcomes in patients with cancer. However, several challenges remain in overcoming the shortcomings of OVT, such as its efficacy, which is still limited and varies between patients. In some cases, the immune response can recognize the OVs and neutralize the viruses before they reach the target tumors, and the virus can evade both the target and immune system, causing inflammation elsewhere, which is also a cause of concern. Additionally, with respect to certain OVs, collateral damage to normal cells can occur, which may lead to side effects and adverse events.

Though the advancement in oncolytic virotherapy is constantly upgraded with new technologies incorporated and proving the effectiveness, several challenges are to be overcome, such as a better understanding of the factors that hinder the viral replication in the TME, the exact mechanism by which one can avoid premature immunogenic viral neutralization and the lack of knowledge on how OVs mediate systemic anti-tumor immune recognition. The safety and ease of administration makes OV an ideal treatment option for cancer patients where other therapies fail. Further knowledge of the mechanism of the therapeutic activity, biomarker identification, and clinical trials with combination therapies will advance the therapeutic application of OV for various cancers.

The therapeutic efficacy of oncolytic viruses may be hindered by a number of factors, despite the fact that they are now being thoroughly studied with various technologies of additions and modifications for targeting various cancers. Significant physical barriers that can prevent viral infection are imposed by the complex tumor microenvironment, which includes dense extracellular matrix composition, reduced tissue vascularization, high interstitial fluid pressure, hypoxia, metabolic acidosis, and tumor necrosis. The delivery method that is effective in treating the tumor is another crucial area of specialization that requires improvement. Intravenous delivery offers a more efficient route for extensive viral dissemination and access to distant tumor tissues, whereas intra-tumoral delivery may avoid the immune system and the peripheral blood barriers. The understanding of these difficulties has led to the development of various innovative techniques to address them, which has improved the therapeutic activity of oncolytic viruses.

Like T-Vec, intratumoral injection administered in a tumor lesion does not spread away from the infected tumor lesion. It could be preferable to administer OV systemically in cases of metastatic disease so that it has the potential to invade all tumor lesions. There is significant genetic, immunologic, and tumor heterogeneity within each patient. Also, tumors evolve over time in response to physiological factors and medication, posing major obstacles to therapies. OV targeting is also affected by tumor heterogeneity, particularly when it is based on particular genetic mutations, cell surface markers, or through transcription-controlled sequences.

There are currently no biomarkers for patient- or cancer-specific OV sensitivity or patient response to treatment with immunovirotherapy. This is essential for improving preclinical models and identifying possible candidates for therapeutic studies, as well as to target clinical therapy to the appropriate patients and identify patients who responds to the treatment during the early course of treatment.

Recent advancements in novel OVs for cancer immunotherapy have demonstrated their potential in commercial products and clinical studies. OVTs have significant potential to be used on an extended range of malignancies that can benefit from immunotherapy and complement current immunotherapeutic treatments. New technologies with complex treatments that are now being extensively developed will overcome the limitation of access for some patients, thereby providing a promising approach for OVT application. Immunotherapy aims for a sustainable full response or recovery, not merely an increase in progression-free or overall survival; thus, we need to prioritize various OVs and develop methods to choose the most promising ones for translation.

## Figures and Tables

**Table 2:** List of OVs approved for cancer immunotherapy.

Commercial Name	Virus	Commercialization Year	Cancer Type	Modifications	Country
Rigvir (ECHO-7)	Picornavirus	2004	Melanoma	Unmodified	Latvia
Oncorine (H101)	Adenovirus Serotype 5	2005	Head and neck cancer	E1B-55K deletion and partial E3 deletion	China
T-VEC (Imlygic)	HSV-1	2015	Melanoma	Deletion of ICP34.5 and ICP47; encoding two copies of human GMCSF	US and Europe
DELYTACT (Teserpaturev/G47∆)	HSV-1	2021	Malignant glioma	Multiple Modification (Deletion of ICP34.5, ICP6 and α47 genes)	Japan

**Table 3:** Summary of OVs used in clinical trials for cancer immunotherapy.

Virus	Trial Name/Number	Modification Strategies	Route of Administration	Combination Strategies	Targeted Therapy	Phase/Status	Ref
HSV	T-VEC/NCT04427306	Insertion: GM-CSF; Deletion: ICP34.5 & ICP47	I.T	None	Melanoma	Phase 2/Recruiting	[176]
	OrienX010/NCT04206358	Insertion: GM-CSF; Deletion: ICP34.5 & ICP47	I.T	JS001	Melanoma	Phase 1/Recruiting	[177]
	OH2/NCT04637698	Insertion: GM-CSF	I.T	None	Pancreatic cancer	Phase 2/Recruiting	[178]
	G207/NCT04482933	Deletion: ICP34.5; Disruption: UL39	I.T	None	Glioma, Astrocytoma, Glioblastoma	Phase 2/Not yet recruiting	[179]
	HF10/NCT03259425	Deletion: UL56, single copy of UL52	I.T	Nivolumab	Melanoma	Phase 2/Terminated	[180]
	SEPREHVIR(HSV1716)/NCT02031965	Deletion: ICP34.5	I.T	None	High-grade glioma	Phase 1/Terminated	[181]
VV	ASP9801/NCT03954067	Encoding: IL-7 and IL-12	I.T	Pembrolizumab	Solid tumors, metastatic cancer, and advanced cancers	Phase 1/Recruiting	[182]
	PexaVec (JX594)/NCT02977156	Insertion: GM-CSF; Deletion: TK	I.T	Ipilimumab	Metastatic cancers and advanced cancers	Phase 1/Recruiting	[183]
	RGV004/NCT04887025	Encoding CD3/CD19 bispecific antibody	I.T	None	B cell lymphoma	Phase 1/Active, not yet recruiting	[184]
	GL-ONC1/NCT02759588	Deletion: A56R, F14.5 L, and J2R	I.P	Chemotherapy and bevacizumab	Fallopian tube cancer, peritoneal carcinomatosis, ovarian cancer	Phase 1/2/Active, not recruiting	[185]
Ad	Colo-Ad1/NCT02053220	T-SIGn	I.T and I.V	None	Bladder cancer, renal cell carcinoma, non-small-cell lung cancer, colon cancer	Phase 1/Completed	[186]
	NG-350A/NCT03852511	Agonistic CD40 antibody Expression	I.V	None	Metastatic epithelial tumor	Phase 1/Recruiting	[187]
	LOAd703/NCT02705196	Encoding TMZ-CD40L and 4-1BBL	I.T	Nab-paclitaxel	Pancreatic cancer	Phase 2/Recruiting	[188]
	H101/NCT04771676	Deletion: E1B, Partial E3	I.P	Chemotherapy	Refractory malignant ascites	Phase 2/Recruiting	[189]
	VCN-01/NCT03284268	Increase in dose for VCN-01	I.T	None	Retinocytoma (recurrent)	Phase 1/Recruiting	[190]
	NG-641/NCT04053283	Expresses fibroblast activation protein (FAP)	I.V	None	Metastatic epithelial tumor	Phase 1/Recruiting	[191]
	ProstAtak/NCT02768363	Insertion: TK	IPOT	Valacyclovir	Prostate cancer	Phase 2/Active, not recruiting	[192]
	DNX-2401/NCT03178032	Insertion: Δ24-RGD	I.A	Surgery	Gliosarcoma, glioma, astrocytoma, glioblastoma	Phase 1/2/Active, not recruiting	[193]
	CG0070/NCT02365818	GM-CSF and Precise promoter	IVES	Immune checkpoint modulation	Bladder cancer	Phase 2/Active, not recruiting	[194]
	Onyx-015/NCT00006106	Deletion: E1B; chimera: Type 2/5	I.T	Cisplatin, fluorouracil	Oral cancer, head and neck cancer, oropharyngeal cancer, lip cancer	Phase 1/Withdrawn	[195]
	Oncos-102/NCT03514836	Insertion: Δ24-RGD-GM-CSF	I.T	DCVAC/PCa\(phase 1), cyclophosphamide (phase 2)	Prostate cancer	Phase 1/2/Terminated	[196]
MV	MV-NIS/NCT03456908	Carcinoembryonic antigen and thyroidal NIS Expression	I.P	F-18 TFB	Endometrial neoplasms, myeloma	Phase 1/Completed	[197]
	MV-s-NAP/NCT04521764	*H. pylori* neutrophil activating protein Expression	I.T	None	Breast carcinoma	Phase 1/Recruiting	[198]
Reovirus	Reolysin/NCT04445844	Yeast cytosine deaminase (Toca511) expression	I.V	Retifanlimab	Breast cancer	Phase 2/Recruiting	[199]
CSV	Cavatak (CVA21)/NCT02316171	Coxsackie viruses A21 (W.T)	IVES	Mitomycin C	Non-muscle-invasive bladder cancer	Phase 1/Completed	[200]

Abbreviations: HSV, herpes simplex virus; VACV, vaccinia virus; AdV, adenovirus; MeV, measles virus; CSV, coxsackie virus; I.T, intra-tumoral; IP, intra-peritoneal; IVES, intra-vesicular; I.V, intravenous; I.POT, intra-prostatic; I.A, intra-arterial; GM-CSF, granulocyte macrophage-colony stimulating factor; TK, thymidine kinase; ICP, infected cell protein; FAP, fibroblast activation protein; T-SIGn, tumor-specific immuno-gene therapy; IL, interleukin; TFB, tetrafluoroborate; W.T, wild type.

## Data Availability

All data needed to support the conclusions are presented in this paper. Additional data related to this study can be obtained from the authors.
